# Electrochemical inhibition bacterial sensor array for detection of water pollutants: artificial neural network (ANN) approach

**DOI:** 10.1007/s00216-019-01853-8

**Published:** 2019-06-03

**Authors:** Hisham Abu-Ali, Alexei Nabok, Thomas J. Smith

**Affiliations:** 1 Materials and Engineering Research Institute, Sheffield Hallam University, City Campus, Paternoster Row, Sheffield, S1 1WB UK; 2 Faculty of Science, Department of Biology, University of Basrah, Basrah, 61004 Iraq; 3 Biomolecular Research Centre, Sheffield Hallam University, City Campus, Sheffield, S1 1WB UK

**Keywords:** Electrochemical sensor array, Inhibition biosensor, Immobilized bacteria, Water pollution, Pattern recognition, Artificial neural network (ANN)

## Abstract

This work reports on further development of an inhibition electrochemical sensor array based on immobilized bacteria for the preliminary detection of a wide range of organic and inorganic pollutants, such as heavy metal salts (HgCl_2_, PbCl_2_, CdCl_2_), pesticides (atrazine, simazine, DDVP), and petrochemicals (hexane, octane, pentane, toluene, pyrene, and ethanol) in water. A series of DC and AC electrochemical measurements, e.g., cyclic voltammograms and impedance spectroscopy, were carried out on screen-printed gold electrodes with three types of bacteria, namely *Escherichia coli*, *Shewanella oneidensis*, and *Methylococcus capsulatus*, immobilized via poly l-lysine. The results obtained showed a possibility of pattern recognition of the above pollutants by their inhibition effect on the three bacteria used. The analysis of a large amount of experimental data was carried out using an artificial neural network (ANN) programme for more accurate identification of pollutants as well as the estimation of their concentration. The results are encouraging for the development of a simple and cost-effective biosensing technology for preliminary in-field analysis (screening) of water samples for the presence of environmental pollutants.

**Electronic supplementary material:**

The online version of this article (10.1007/s00216-019-01853-8) contains supplementary material, which is available to authorized users.

**Figure:**
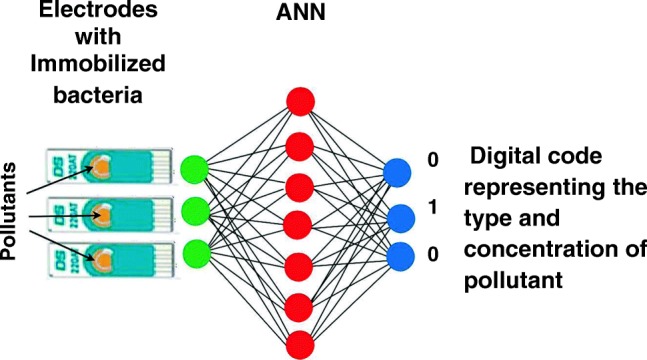
Graphical abstract

## Introduction

Nowadays, water pollution caused by the presence of any chemicals in fresh- and seawater which reduce the water quality and affect living organisms in the aquatic environment has become a major problem associated with areas of intensive industry and agriculture. A significant part of water contamination comes from transport, heavy industry, petrochemical industry, and agricultural activities which release large numbers of toxic chemicals, particularly heavy metals, pesticides, and petrochemicals, in the atmosphere and aquatic environment. Typical petrochemical contaminants are hydrocarbons, alcohols, ketones, benzene derivatives (or BTEX), etc. Considering the adverse effects of the above pollutants on humans, animals, and wildlife, the environmental agencies and World Health Organization set quite low limits (from 0.1 to 0.5 mg/l) for heavy metals (Hg, Pb, and Cd), pesticides (DDT, DDE, TDE, etc.), and some petrochemical (i.e., methyl alcohol and BTEX) pollutants in drinking water, food, and feed [1]. Worldwide environmental legislation clearly indicates the need for reliable environmental monitoring methods, which are also fast, portable, and cost-effective [2]. Therefore, the determination of industrial and agricultural pollutants such as heavy metal ions, hydrocarbons, and pesticides at very low concentration levels is one of the main goals for environmental science nowadays [3].

Conventional analytical techniques such as gas chromatography, inductively coupled plasma mass spectroscopy (ICP-MS), and high-performance liquid chromatography (HPLC) are very sensitive and reliable [4]. However, they suffer from the disadvantages of high cost, time consuming, the need for highly trained technicians, and the fact that they are mostly laboratory bound.

An alternative approach lies in the use of biosensors which present distinct advantages of high specificity, fast response times, portability, ease of use, and low cost [5, 6]. Since the invention of the electrochemical biosensor for glucose detection in 1962, biosensors have been intensively studied and extensively utilized in various applications, ranging from public health and environmental monitoring to homeland security and food safety [7]. Electrochemical methods can be successfully used for simple and rapid detection of the abovementioned pollutants in the aquatic environment [8].

There are two approaches of using biosensors for environmental control: (i) immuno-sensing where pollutants can be bound to highly specific receptors, i.e., antibodies or aptamers, and (ii) inhibition sensors mostly based on enzymes which are inhibited by pollutants [9]. The immuno-sensors, despite their high selectivity and sensitivity of detection, require the production (or synthesis) of a large number of bio-receptors for every pollutant which may come at a cost. The inhibition sensor approach of using a small number of bio-receptors is much simpler and less expensive although it may not be highly sensitive and not particularly selective because the receptors could be affected by different types of pollutants. Therefore, in order to identify the pollutants, several sensors having different bio-receptors have to be used simultaneously which brings the concept of a sensor array and the associated data processing, for example, artificial neural network (ANN) [10].

Instead of extremely fragile and sensitive-to-environment enzymes, whole cells and bacteria can be used as much more robust bio-receptors in inhibition sensors and sensor arrays. Several successful inhibition sensor array developments utilizing whole cells [11, 12] and bacteria [13] were reported recently. Our recent research proved the concept of pattern recognition of water pollutants using optical and electrochemical measurements of both liquid bacterial samples and immobilized bacteria [14–16] and a limited number of pollutants of different types, e.g., heavy metals, pesticides, and petro-chemicals, in rather high concentrations.

In this work, we continue the development of a bacteria-based electrochemical sensor array for detection of a wide range of pollutants including several heavy metals, pesticides, and petrochemicals in low concentrations down to 0.1 μM. The analysis of a large number of data, e.g., the sensor responses of each channel to every pollutant, is carried out using the MatLab ANN programme.

## Experimental methodology

### Preparation of bacterial samples

Three types of bacteria were selected for this work: (i) *Escherichia coli* (*E. coli*, K12 strain), a gram-negative bacterial type generally sensitive to different types of pollutants including heavy metals, pesticides, and hydrocarbons [17]; (ii) *Shewanella oneidensis* (*S. oneidensis* MR-1 strain), gram-negative bacteria known to be tolerant of heavy metals [18]; and (iii) methanotrophic bacteria (*Methylococcus capsulatus* Bath strain), gram-negative bacteria which consume some petrochemicals as nutrients [19, 20]. Luria-Bertani (LB) broth was used as a growth medium for both *E. coli* and *S. oneidensis* bacterial cell cultures [21], while *M. capsulatus* (Bath) were grown in nitrate mineral salts (NMS) medium [22]. The bacterial growth media and phosphate buffer saline (PBS) were acquired from Sigma-Aldrich Co. Other chemicals, i.e., HgCl_2_, PbCl_2_, and CdCl_2_ salts; atrazine, simazine, and DDVP; hexane, octane, pentane, toluene, pyrene, and ethanol; and poly l-lysine (PLl), were also purchased from Sigma-Aldrich Co.

### Bacterial culture growth

All bacterial strains used in this study were provided by the Biomolecular Research Centre, Sheffield Hallam University. All strains were stored at − 70 °C in 15% glycerol to be used as a bacterial source in future. Cultivation of bacteria was carried out in several stages. The first step was to cultivate a specific strain of bacteria in a Petri dish containing solid agar. In the second stage, one colony of bacteria was added into a sterile flask containing 50 ml of liquid LB broth (for *E. coli* and *S. oneidensis*) or NMS medium (for *M. capsulatus*). Finally, the flask containing the bacterial culture was placed inside the incubator operating at 150 rpm shaking speed. The incubation temperature was 30 °C for *Shewanella oneidensis* and *M. capsulatus* (Bath), while 37 °C was used for *E. coli*. Bacterial growth time was 16 h for *E. coli*, 24 h for *Shewanella oneidensis*, and 72 h for *M. capsulatus* (Bath).

### Bacterial sensor array construction

**Fig. 1:**
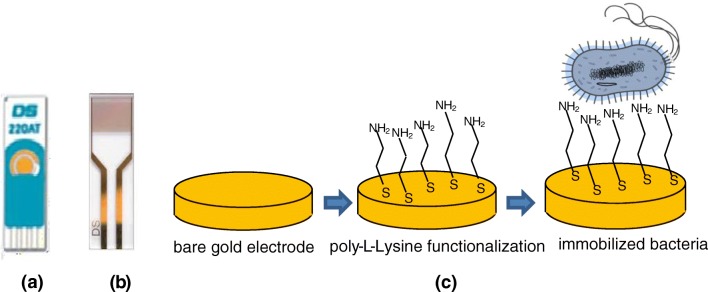
DropSens three-electrode assembly (**a**), DropSens interdigitated electrodes (**b**), schematic diagram of the bacterial immobilization procedure (c)

A bacterial biosensor array (or multi-sensor) consisted of three screen-printed electrodes having different sensing characteristics towards investigated analytes. The sensor array was fabricated by immobilizing the three types of bacterial cells, on the surface of screen-printed gold electrodes via poly l-lysine (PLl) [23] (see Fig. 1). In order to achieve the highest surface coverage, the maximal concentration of bacterial cells from 10 to 30 10^9^ cm^−3^ corresponding to the end of the bacterial exponential growth phase, e.g., 16 h for *E. coli*, 24 h for *S. oneidensis*, and 72 h for *M. capsulatus*, was used for immobilization. For this reason, the surface of gold was treated in a 1:1000 mixture of PLl (0.1 mg/ml) and deionized water for 1 h at 37 °C. Then, bacteria were immobilized by dropping stock solutions of *E. coli*, or *M. capsulatus* (Bath), or *S. oneidensis* on the modified electrodes, incubating for 1 h, and then washing out non-bound bacteria with PBS [15].

### Preparation of analyte solutions

The inhibition effects on the abovementioned bacteria have been studied by exposing them to the following chemicals (pollutants): HgCl_2_, PbCl_2_, and ZnCl_2_ salts; atrazine, simazine, and DDVP; and hexane, pentane, octane, toluene, ethanol, and pyrene. Their solutions of different concentrations (0.1, 1, 10, 100, and 1000 μM) were prepared by multiple dilution of 1 mM stock solution of each analyte dissolved in deionized water. Forty percent ethanol solution in water was used for dissolving the hydrocarbons, toluene, and pyrene. The samples of immobilized bacteria were treated by immersing them in the required solutions of the above chemicals for 2 h.

### Electrochemical experimental measurements

All cyclic voltammogram (CV) electrochemical measurements were carried out on a DropSTAT4000P potentiostat instrument (from DropSens) controlled by Autolab software using DropSens screen-printed gold electrodes (SPGEs); the voltage range from − 0.5 to + 0.5 V and the scan rate of 10 mV/s were used. SPGEs have a conventional three-electrode configuration with gold working and counter electrodes and Ag/AgCl pseudo-reference electrode (see Fig. 1a) [24]. The CV measurements were carried on SPGEs with all three types of bacteria immobilized on the surface of gold electrodes via poly l-lysine (PLl). The CV measurements were carried out in PBS both before and after treatment with (Hg^2+^), (Pb^2+^), and (Cd^2+^) salts, pesticides (atrazine, simazine, and DDVP), and hydrocarbons (hexane, pentane, octane, toluene, pyrene, and ethanol) in different concentrations.

Impedance spectra were measured using an impedance analyzer (4000A EG & G Instrument) and gold interdigitated electrodes (from DropSens) containing 250 fringes on each side spaced by 5 μm, the overlapping length 6.76 mm (see Fig. 1b). The AC voltage amplitude was 5 mV with the frequency varied from 100 mHz to 100 kHz; no DC bias was applied [25]. The use of interdigitated electrodes was recommended by the supplier (DropSens) for impedance measurements. Similarly to CVs, the impedance spectra measurements were carried out on electrodes coated with immobilized bacteria, in buffer solutions containing different pollutant concentrations.

## Electrochemical measurement results

### Cyclic voltammograms

**Fig. 2:**
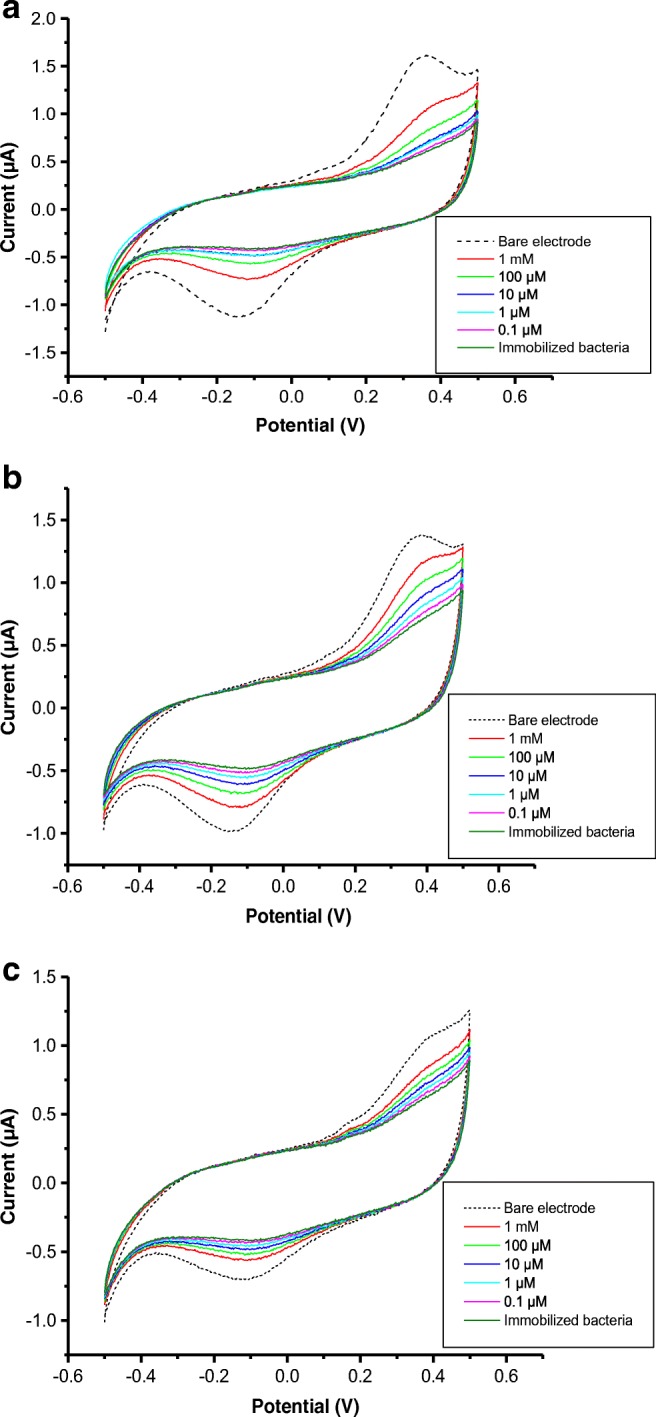
CVs recorded on electrodes with immobilized *E. coli* treated with toluene (**a**), *M. capsulatus* treated with atrazine (**b**), and *S. oneidensis* treated with HgCl_2_ (**c**) in different concentrations

Typical cyclic voltammograms (CVs) in Fig. 2a–c show the increase in both anodic and cathodic currents upon increasing the concentration of pollutants in all three cases presented: the toluene which effects the immobilized *E.coli* (a), simazine effect on *M. capsulatus* (b), and HgCl_2_ effect on *S. oneidensis* (c)*.* Similar results were observed for the effect of all other pollutants used for the respective bacteria.

It should be noted that CVs of uncoated electrodes in Fig. 2 show the characteristic anodic peaks (at about + 0.3 to + 0.4 V) and cathodic peaks (at about − 0.15 V) which are associated with electrochemical reactions in PBS. The bacteria immobilized on the electrode surface act as an insulating layer, thus reducing the current substantially. The observed threefold drop of the DC current after coating the electrodes with bacteria corresponds approximately to 30% bacterial surface coverage. However, the bacteria are damaged by exposure to pollutants and their insulating properties are reduced. This clearly explains the observed behavior. The correlations between the values of the anodic current and pollutant concentrations were therefore established which constitutes the main principle of electrochemical detection of pollutants using bacteria.

### Electrochemical impedance spectroscopy

**Fig. 3:**
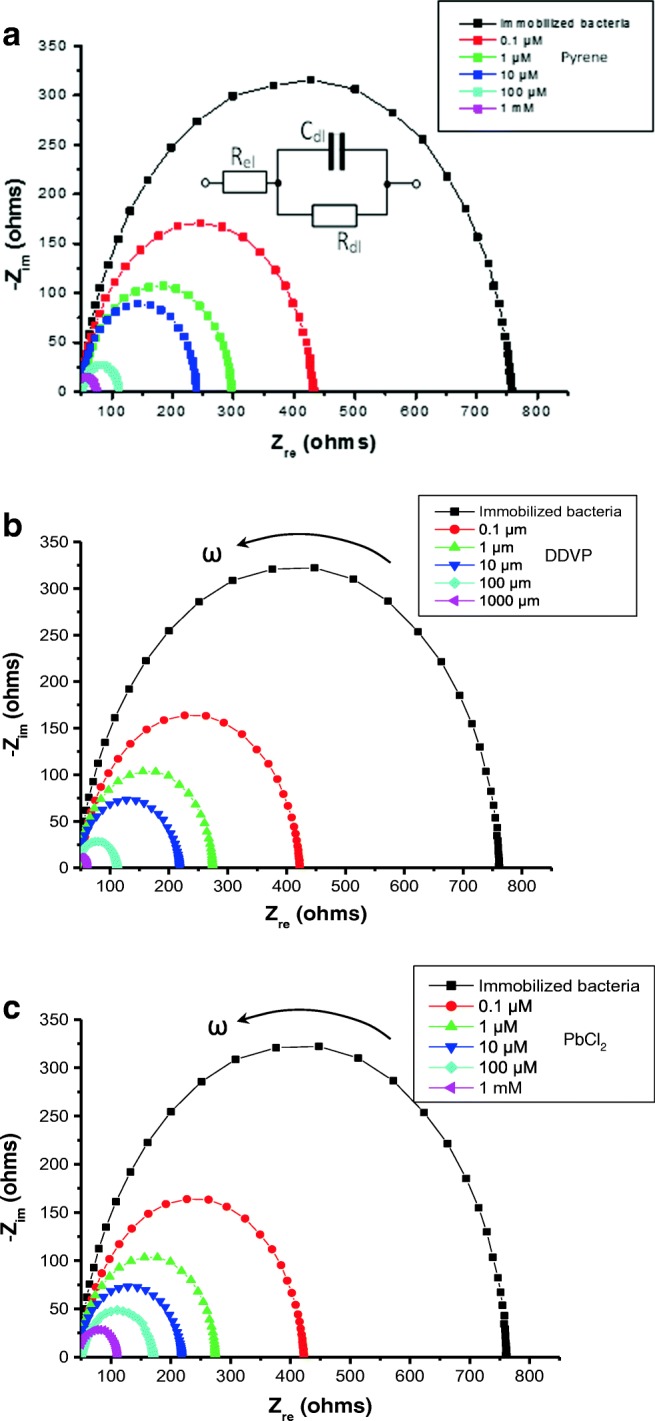
Examples of Nyquist plots (− *Z*_im_ vs *Z*_re_) for interdigitated electrodes with immobilized bacteria exposed to different pollutants of different concentrations: *E. coli* treated with pyrene (**a**), *M. capsulatus* treated with DDVP (**b**), and *S. oneidensis* treated with PbCl_2_ (**c**); the equivalent circuit is shown as an inset in **a**; the arrows indicate the direction of frequency increase

The electrical properties of bacteria immobilized on metal electrodes were studied with electrochemical impedance spectroscopy. Typical results of this study are presented as Nyquist plots in Fig. 3. As one can see, for all three graphs selected which correspond to three bacteria, the exposure to pollutants caused both the reduction in size of semi-circles and their shift to low resistance values which is an indication of the decrease in the double-layer resistance.

More detailed analysis of the impedance spectra could be done using a simplified equivalent circuit diagram (shown as an inset in Fig. 3a) which consists of a connected-in-parallel resistor *R*_*db*_ and capacitor *C*_*db*_ of an electrical double layer on the electrode surface and the resistor of the electrolyte *R*_*el*_ connected in series. The negative sign of the imaginary part of the impedance indicated its capacitive character.

According to the theory [26], at high frequencies (*ω* → ∞), the real part of impedance *Z*_*re*_ *= R*_*el*_, while at low frequencies (*ω* → 0), *Z*_*re*_ *= R*_*el*_ *+ R*_*db*_ *≈ R*_*db*_ because *R*_*el*_ is usually quite small *R*_*el*_ *<< R*_*db*_. The reduction on the values of *R*_*db*_ as a result of bacterial exposure to pollutants is apparent from the impedance spectroscopy data presented in Fig. 3. This confirms the previously observed facts that bacteria are losing their insulating properties upon exposure to pollutants.

**Fig. 4:**
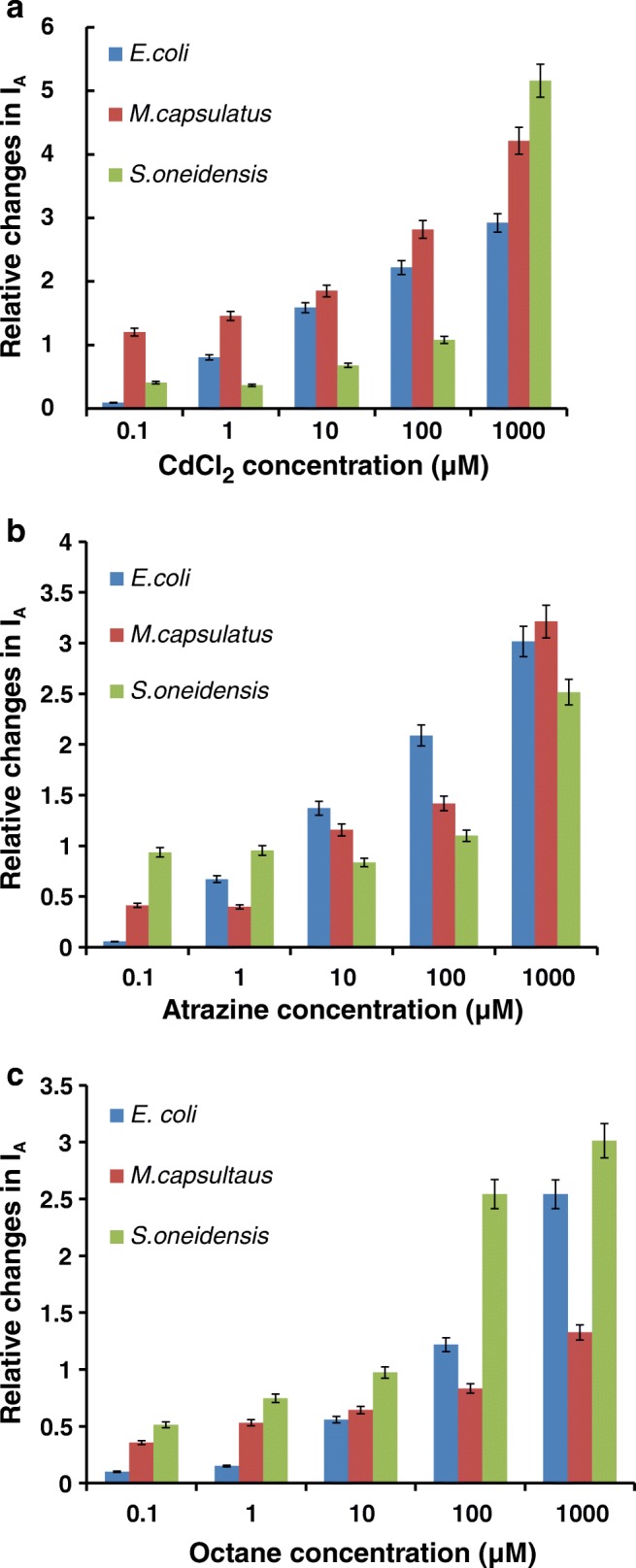
The dependence of relative changes of the anode at + 0.5 V for immobilized *E. coli*, *M. capsulatus*, and *S. oneidensis* on the concentration of CdCl_2_ (**a**), atrazine (**b**), and octane (**c**)

More detailed analysis of the effect of pollutants was carried out by presenting the data of CV measurements as the dependence of the anodic current at + 0.5 V against the pollutant concentration; such dependences are shown in Fig. 4. The comparison of the effect of different pollutants on the three types of bacteria used has to be performed using the relative changes of anodic current (Δ*I*_A_) normalized by the reference current *I*_A0_ corresponding to the CVs recorded on electrodes with immobilized bacteria in PBS without addition of pollutants:

$$ \varDelta {I}_{\mathrm{A}}/{I}_{\mathrm{A}0}=\left({I}_{\mathrm{A}}-{I}_{\mathrm{A}0}\right)/{I}_{\mathrm{A}0} $$

As was concluded from the impedance spectra data, the effect of the pollutants (even metal salts) on the total resistance (and thus the current) is negligibly small.

As one can see from Fig. 4a, the effects of CdCl_2_ on *S. oneidensis*, *M. capsulatus*, and *E. coli* are completely different: *∆I*_A_/*I*_A0_ goes up with the increase in CdCl_2_ concentration for *E. coli* and *M. capsulatus* which means that both *E. coli* and *M. capsulatus* bacteria are inhibited by Cd^2+^ ions and becoming less electrically resisting, while *∆I*_A_/*I*_A0_ is almost flat at low concentrations of CdCl_2_ and slightly increases at a high concentration of 1 mM. This means that *S. oneidensis* are practically not affected by CdCl_2_ in low concentrations but inhibited at high concentrations. Similarly, in Fig. 4b, atrazine inhibits *E. coli* and *M. capsulatus*, while its effect on *S. oneidensis* is more or less independent on atrazine concentration (excluding the high concentration of 1 mM). According to data in Fig. 4c, octane inhibits *E. coli* and *S. oneidensis*, but has a much smaller inhibition effect on *M. capsulatus*. The above results are very promising and demonstrate a possibility of pattern recognition of pollutants using a bacterial sensor array.

The reproducibility of CV and EIS measurements is reasonably good, e.g., within 10% of current and impedance values. Stability of the samples with immobilized bacteria was studied, and it was found that the storage of samples with immobilized bacteria for 24 h in the fridge at 4 °C has no effect on sensor responses. The activity of bacteria is, however, reduced by 10–15% after 48 h storage and further down to 50% after 72 h storage.

## Sensor array data analysis

### Identification of water pollutants using pseudo-3D graphs of sensor responses

**Fig. 5:**
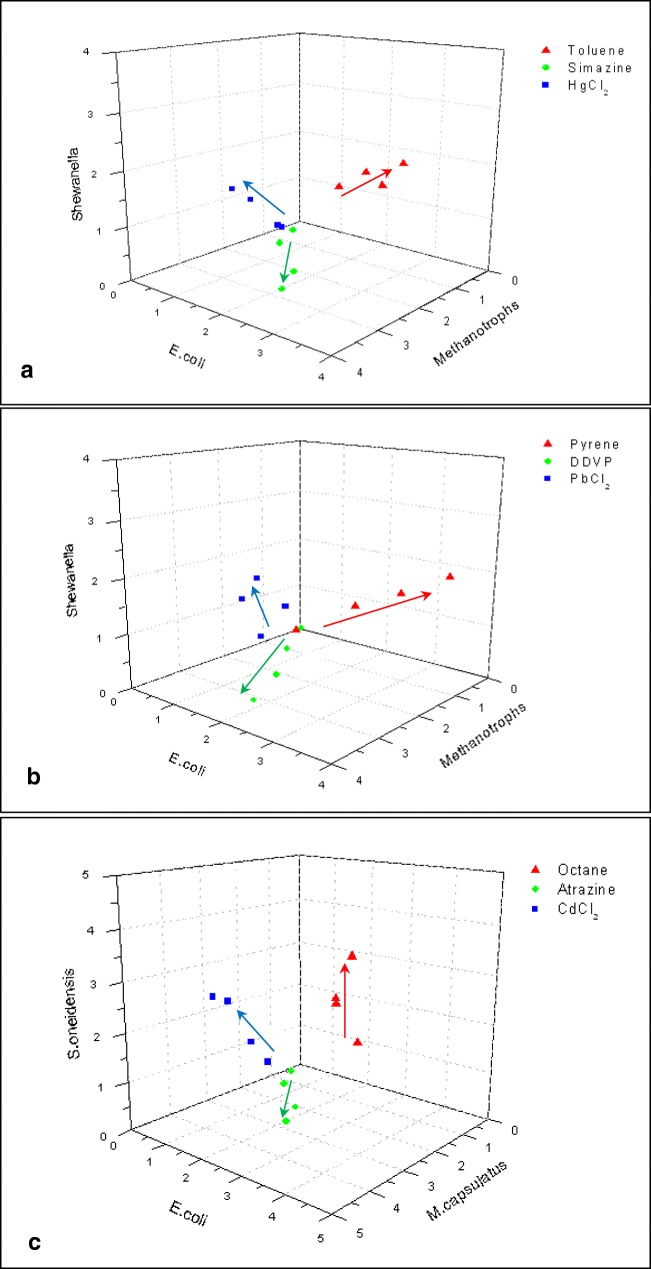
3D graphs of responses of three sensing channels (having different bacteria) to different pollutants: toluene, simazine, and HgCl_2_ (**a**); pyrene, DDVP, and PbCl_2_ (**b**); and octane, atrazine, and CdCl_2_ (**c**) in different concentrations from 0.1 to 100 μM. Arrows indicate the directions of concentration increase

All the results obtained from the sensor array containing three electrodes functionalized by three different types of bacteria, namely *E. coli*, *S. oneidensis*, and *M. capsulatus*, treated by 12 different pollutants, e.g., heavy metal ions (Hg^2+^, Pb^2+^, and Cd^2+^), pesticides (atrazine, simazine, and DDVP), and petrochemicals (hexane, octane and pentane, toluene, pyrene, and ethanol) of different concentrations of 0.1, 1, 10, 100, and 1000 μM, were analyzed first using pseudo-3D plots of sensor responses in Fig. 5.

Figure 5a is a simple 3D graph of relative changes of the anodic current (Δ*I*_A_/*I*_A0_) of three channels corresponding to three bacterial types (*Escherichia coli*, *Methylococcus capsulatus* (Bath), and *Shewanella oneidensis*) in response to exposure to toluene, simazine, and HgCl_2_ in different concentrations (the data points for the largest concentrations (1 μM) of pollutants were excluded because of their adverse effect on all three bacteria). Similar 3D graphs are presented in Fig. 5b for pyrene, DDVP, and PbCl_2_ and in Fig. 5c for octane, atrazine, and CdCl_2_. The data in Fig. 5 demonstrate a clear separation of responses for heavy metal ions (blue dots which appear on the left side of the graphs), pesticides (green dots which appear at the bottom part), and petrochemicals (red dots which appear on the right side). It can be concluded that a simple pseudo-3D graph of the three sensor responses allows the classification of pollutants studied into three groups, e.g., heavy metals, pesticides, and petrochemicals, which demonstrate clearly the principles of pattern recognition. Further identification of pollutants within each group, for example, distinguishing between Hg, Pb, and Cd ions, is difficult to achieve using such a simple approach because all the data points for heavy metals appeared in the same section of 3D space in Fig. 5. Precise identification of pesticides and petrochemicals faces the same difficulties. It is also difficult to estimate the concentration of pollutants because of random scattering of data points.

### ANN data analysis of water pollutants

**Fig. 6:**
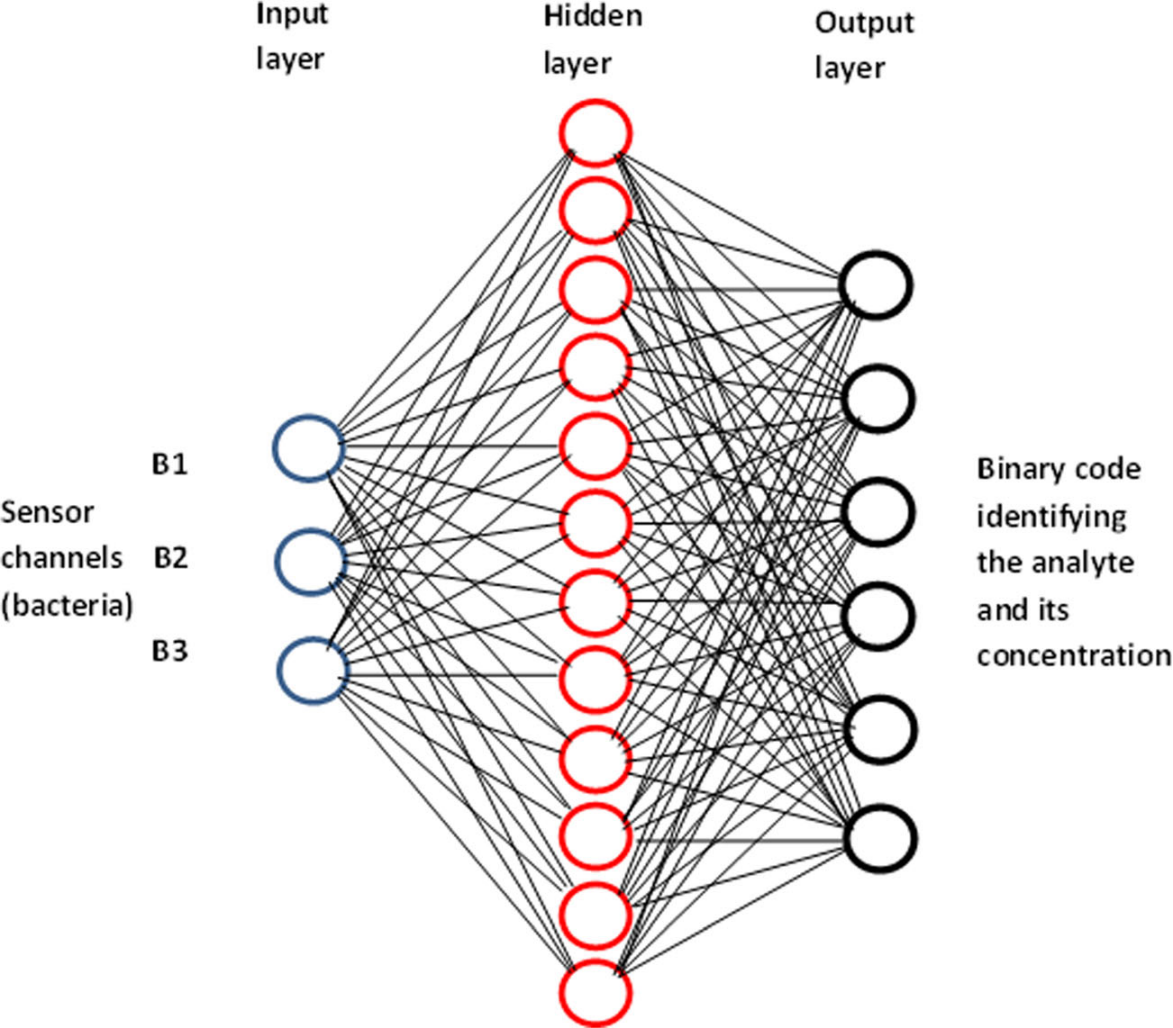
ANN architecture for classification of pollutants and estimation of their concentration

Much more accurate recognition of pollutants was achieved with the use of the artificial neural network (ANN) programme written using Neural Network Toolbox, version 4.0, within MATLAB 6.1 (Mathworks, Natick, MA). The ANN model, shown in Fig. 6, consists of three layers: (i) the input layer of the responses of three sensing channels (containing different bacteria); (ii) the hidden layer containing 12 neurons corresponding to 12 pollutants studied; and (iii) the output layer representing a six-digit binary code which identifies the type of pollutants and their concentration.

The designed ANN programme is therefore capable of identification of pollutants as well as the rough estimation of their concentration by rounding the output to the nearest quantized concentration value, e.g., 0.1 μM, 1 μM, 10 μM, 100 μM, and 1 mM.

The ANN was trained by multiple feeding the experimental results, e.g., the responses of all three sensing channels to all 12 pollutants (Hg^2+^, Pb^2+^, Cd^2+^, atrazine, simazine, DDVP, hexane, pentane, pyrene, toluene, octane, and ethanol) in five concentrations (0.1 μM, 1 μM, 10 μM, 100 μM, and 1 mM). The training data are presented in Table S1 in the Electronic Supplementary Material (ESM). This table also contains the identification digital codes. The ANN training procedure exploited the Levenberg-Marquardt algorithm to optimize the weights of neurons in a hidden layer. This algorithm appeared to be the fastest method for the network training using the limited experimental data of this study. A hyperbolic tangent was used as the activation function for the hidden neurons, and a log-sigmoid function was used for the output neurons. The training was performed for 250,000 epochs (e.g., 250,000 repetitions of data feeding) with the mean square error (MSE) goal set to 10^−10^. Figure S1 in ESM shows the saturation of MSE at about 250,000 epochs.

After the training, the ANN programme was tested by feeding the data obtained from the bacterial sensor array for PBS solutions spiked with a particular concentration of pollutants randomly selected within the 1–100-μM concentration range. The results of such tests are summarized in Table 1. The ANN outcome is a six-digit code representing the type of pollutant and its concentration rounded to the nearest quantized concentration value.

**Table 1:** The results of ANN identification of pollutants and estimation of their concentration

Input values			Output values								
*E. coli*	*M. capsulatus*	*S. oneidensis*	Binary code						Pollutant	Concentration (μM)	
										Obtained	Actual
0.09417	0.58032	0.06211	0	0	0	0	1	0	Hg^2+^	1	0.66
0.46138	1.48214	1.08641	0	0	0	0	1	1		10	1.3
1.19823	1.37532	1.65302	0	0	0	1	0	0		100	66
0.09023	0.45121	0.67656	0	0	0	1	1	1	Pb^2+^	1	1.25
0.48352	1.56176	0.87403	0	0	1	0	0	0		10	22
1.21501	1.71608	2.47263	0	0	1	0	0	1		100	83
0.54321	0.59745	1.31435	0	0	1	1	0	0	Cd^2+^	1	1.37
0.76232	1.13421	1.54623	0	0	1	1	0	1		10	26
1.55412	1.66534	1.82152	0	0	1	1	1	0		100	76
0.55657	1.01232	0.01566	0	1	0	0	0	1	Atrazine	1	1.45
0.30508	2.09684	0.08508	0	1	0	0	1	0		10	24
1.06593	2.95676	0.97593	0	1	0	0	1	1		100	71
0.76231	1.20342	0.18784	0	1	0	1	1	0	Simazine	1	1.61
1.54231	2.47652	0.36941	0	1	0	1	1	1		10	12.5
2.14325	2.89732	0.89876	0	1	1	0	0	0		100	87
0.50453	1.13423	0.05566	0	1	1	0	1	1	DDVP	1	1.1
1.65483	2.08563	0.02508	0	1	1	1	0	0		10	13.8
2.26314	2.87342	0.09593	0	1	1	1	0	1		100	99
0.03290	1.24231	0.49265	1	0	0	0	0	0	Hexane	1	1.2
1.04398	2.72134	0.56021	1	0	0	0	0	1		10	32
2.89296	2.36532	0.75134	1	0	0	0	1	0		100	83
0.02965	1.299123	0.27365	1	0	0	1	0	1	Octane	1	1.22
0.95120	2.61860	0.41931	1	0	0	1	1	0		10	17
2.00623	2.92861	0.65108	1	0	0	1	1	1		100	77
0.71525	1.47123	0.51132	1	0	1	0	1	0	Pentane	1	1.6
1.29723	2.68243	0.74167	1	0	1	0	1	1		10	18
2.26211	2.85412	0.97133	1	0	1	1	1	0		100	89
0.90123	2.51252	0.46326	1	0	1	1	1	1	Toluene	1	1.2
2.03251	2.85421	0.59131	1	1	0	0	0	0		10	11
1.98589	2.85862	2.87109	1	1	0	0	0	1		100	93
0.80173	1.01099	0.38134	1	1	0	1	0	0	Pyrene	1	1.4
2.01351	1.13209	0.53965	1	1	0	1	0	1		10	11.6
2.90598	1.07213	1.43312	1	1	0	1	1	0		100	83
0.15432	0.51974	0.63144	1	1	1	0	0	1	Ethanol	1	1.5
0.48453	1.53342	0.54762	1	1	1	0	1	0		10	12.1
1.31541	1.81653	2.65021	1	1	1	0	1	1		100	97

Despite the limited amount of data for ANN training, the programme managed to identify the pollutants correctly. The comparison of values in the last two columns representing, respectively, the obtained and actual concentrations of pollutants showed that the concentration was estimated correctly. For example, the sample spiked with 1.45 μM of atrazine was identified by binary code 010001 which corresponds to atrazine in a concentration of 1 μM; the sample spiked with 0.66 μM of HgCl_2_ was identified by code 000001 corresponding to Hg^2+^ in a concentration of 1 μM; the sample spiked with 83 μM of pyrene was identified by code 110110 as pyrene in a concentration of 100 μM.

The results obtained are very promising since simple electrochemical measurements of the anodic current at + 0.5 V combined with ANN-based data processing allow both the identification of the pollutants studied and a rough estimation of their concentrations.

## Conclusions and future work

A series of electrochemical measurements, e.g., cyclic voltammograms and impedance spectra, on screen-printed electrodes with immobilized bacteria proved the concept of using bacteria as bio-receptors in the inhibition sensor array. All three bacteria studied, *E. coli*, *M. capsulatus*, and *S. oneidensis*, immobilized on the electrodes appeared to act as insulators and reduced the charge transfer. The inhibition effect of 12 pollutants studied lies in the reduction of bacterial electrical resistivity. The inhibition effect depends on the type of bacteria, the type of pollutant, and their concentration which provides an opportunity for pattern recognition of pollutants using simple and illustrative pseudo-3D plots of sensor responses. The use of ANN software for data processing allowed the more accurate identification of water pollutants, e.g., heavy metals, pesticides, and hydrocarbons as well as the estimation of their concentration in the range from 0.1 μM to 1 mM.

The developed electrochemical inhibition sensor array based on bacteria immobilized on the surface of screen-printed electrodes proved to be a useful analytical tool for water pollution analysis. Such a biosensor array may fit the purpose of preliminary testing (or screening) of water samples. The samples identified by the sensor array as “contaminated” by a particular pollutant in a certain concentration range can be passed to specialized laboratories for further more detailed and more accurate testing. In this way, both the time and cost of analysis of water samples could be substantially reduced.

Future work which is currently underway focuses on extending the range of pollutants, the improvement of the ANN data processing, and the testing of real water samples. The ANN software should be able to identify pollutants in complex samples (including real samples of water from different sources) containing a mixture of pollutants. The evaluation of pollutant concentration should be also more precise; for that purpose, a separate ANN programme employing a polynomial approximation of concentration dependencies of sensor responses has to be designed.

## Electronic supplementary material

ESM 1 (PDF 141 kb)
