# Supplementary material for: Electrochemical inhibition bacterial sensor array for detection of water pollutants: artificial neural network (ANN) approach
Source: Anal Bioanal Chem. 2019 Jun 3;411(29):7659–68. doi: 10.1007/s00216-019-01853-8 (PMC6881469; doi:10.1007/s00216-019-01853-8)
Supplement: Supplementary file 1 — (PDF 141 kb) [file 216_2019_1853_MOESM1_ESM.pdf]

Analytical and Bioanalytical Chemistry

Electronic Supplementary Material

Electrochemical inhibition bacterial sensor array for detection of water pollutants: artificial neural network (ANN) approach

Hisham Abu-Ali, Alexei Nabok, Thomas J. Smith

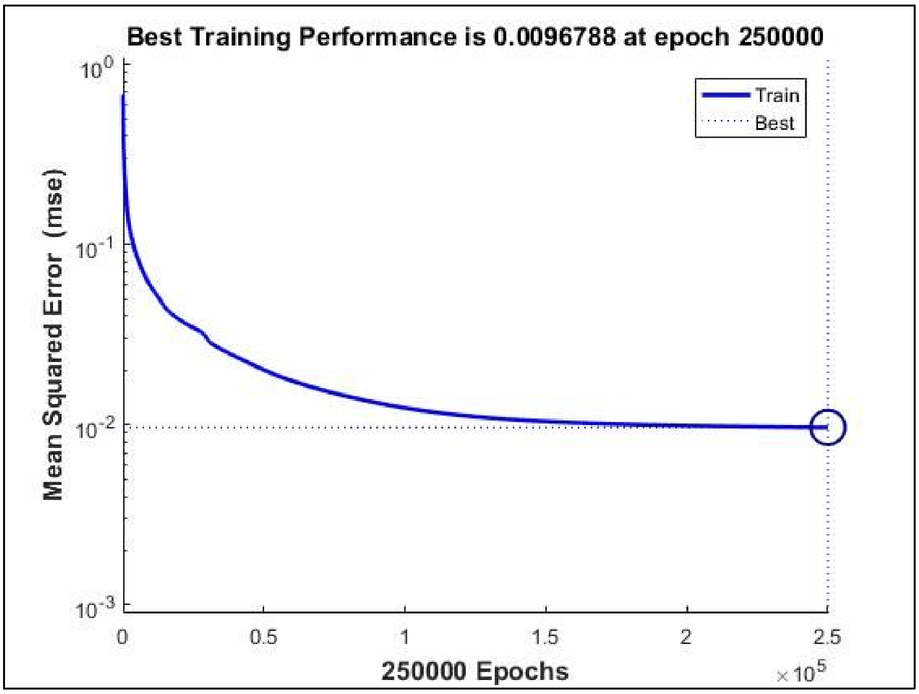

Fig. S1 ANN training: Reduction of MSE during the 250,000 epochs of data feeding

Table S1 Data set of the neural network training

C μM	Input values			Output values						
	E.coli	*M.capsulatus*	*S.oneidensis*	*Binary code*						*target analyte*
**0.1**	0.0941	0.2011	0.0566	0	0	0	0	0	1	**Hg^{2+}**
**1**	0.8061	0.6568	1.0651	0	0	0	0	1	0	
**10**	0.5862	1.5508	1.0790	0	0	0	0	1	1	
**100**	1.2180	1.4181	1.7796	0	0	0	1	0	0	
**1000**	1.9207	0.2134	2.1583	0	0	0	1	0	1	
**0.1**	0.1015	0.3573	0.5132	0	0	0	1	1	0	**Pb^{2+}**
**1**	0.1513	0.5321	0.7467	0	0	0	1	1	1	
**10**	0.5591	1.6432	0.9733	0	0	1	0	0	0	
**100**	1.2180	1.8324	2.5427	0	0	1	0	0	1	
**1000**	2.5423	2.3265	3.0132	0	0	1	0	1	0	
**0.1**	0.3242	0.3423	0.1098	0	0	1	0	1	1	**Cd^{2+}**
**1**	0.5342	0.7621	1.2765	0	0	1	1	0	0	
**10**	0.7454	1.2310	1.6546	0	0	1	1	0	1	
**100**	1.6454	1.5231	1.9342	0	0	1	1	1	0	
**1000**	2.5534	0.8702	2.3287	0	0	1	1	1	1	
**0.1**	0.0557	0.0123	0.0357	0	1	0	0	0	0	**atrazine**
**1**	0.6705	1.0168	0.0551	0	1	0	0	0	1	
**10**	0.3716	2.1568	0.0359	0	1	0	0	1	0	
**100**	1.0880	3.4181	0.1996	0	1	0	0	1	1	
**1000**	2.0158	4.2134	2.5153	0	1	0	1	0	0	
**0.1**	0.2623	0.1034	0.1078	0	1	0	1	0	1	**simazine**
**1**	0.8423	1.3765	0.2094	0	1	0	1	1	0	
**10**	1.6433	2.5473	0.4988	0	1	0	1	1	1	
**100**	2.1653	3.0231	0.9199	0	1	1	0	0	0	
**1000**	3.5432	4.7843	3.0742	0	1	1	0	0	1	
**0.1**	0.2045	0.5342	0.0357	0	1	1	0	1	0	**DDVP**
**1**	0.6548	1.2856	0.0551	0	1	1	0	1	1	
**10**	1.7231	2.1234	0.0359	0	1	1	1	0	0	
**100**	2.3075	3.0562	0.1996	0	1	1	1	0	1	
**1000**	3.2415	4.5324	2.5153	0	1	1	1	1	0	
**0.1**	0.0266	1.0912	0.4366	0	1	1	1	1	1	**hexane**
**1**	0.0351	1.1861	0.5931	1	0	0	0	0	0	

C	Input values			Output values						
**10**	1.0598	2.5862	0.8109	1	0	0	0	0	1	
**100**	2.9896	3.2180	0.9488	1	0	0	0	1	0	
**1000**	3.1583	4.9207	5.1583	1	0	0	0	1	1	
**0.1**	0.0266	1.1099	0.2834	1	0	0	1	0	0	**octane**
**1**	0.0351	1.3209	0.3965	1	0	0	1	0	1	
**10**	1.0598	2.7213	0.5234	1	0	0	1	1	0	
**100**	1.9896	3.1653	0.8534	1	0	0	1	1	1	
**1000**	2.1583	3.5202	3.0652	1	0	1	0	0	0	
**0.1**	0.5432	1.1974	0.3144	1	0	1	0	0	1	**pentane**
**1**	0.8453	1.5342	0.5462	1	0	1	0	1	0	
**10**	1.3541	2.8653	0.7451	1	0	1	0	1	1	
**100**	2.2761	3.6452	0.8953	1	0	1	1	0	0	
**1000**	3.4532	4.2756	4.3523	1	0	1	1	0	1	
**0.1**	0.0350	1.32091	0.3965	1	0	1	1	1	0	**toluene**
**1**	1.0598	2.72134	0.5234	1	0	1	1	1	1	
**10**	1.9896	3.16532	0.8534	1	1	0	0	0	0	
**100**	2.1583	3.52016	3.0652	1	1	0	0	0	1	
**1000**	0.0912	1.20111	0.4065	1	1	0	0	1	0	
**0.1**	0.2965	0.99123	0.7365	1	1	0	0	1	1	**pyrene**
**1**	0.9050	1.1860	0.4931	1	1	0	1	0	0	
**10**	2.0598	1.2861	0.6108	1	1	0	1	0	1	
**100**	2.9896	1.0180	1.5487	1	1	0	1	1	0	
**1000**	4.1583	0.9206	5.1583	1	1	0	1	1	1	
**0.1**	0.1015	0.3573	0.5132	1	1	1	0	0	0	**ethanol**
**1**	0.1513	0.5321	0.7467	1	1	1	0	0	1	
**10**	0.5591	1.6432	0.9733	1	1	1	0	1	0	
**100**	1.2180	1.8324	2.5426	1	1	1	0	1	1	
**1000**	2.5423	2.3265	3.0132	1	1	1	1	0	0	
